# Sequential Administration of SARS-CoV-2 Strains-Based Vaccines Effectively Induces Potent Immune Responses against Previously Unexposed Omicron Strain

**DOI:** 10.3390/pathogens12050655

**Published:** 2023-04-28

**Authors:** Qianying Wang, Shuhui Wang, Ying Liu, Shuo Wang, Hong Peng, Yanling Hao, Kunxue Hong, Dan Li, Yiming Shao

**Affiliations:** State Key Laboratory for Infectious Disease Prevention and Control, National Center for AIDS/STD Control and Prevention, Chinese Center for Disease Control and Prevention, Beijing 102206, China; wangqianyingchn@foxmail.com (Q.W.);

**Keywords:** SARS-CoV-2, vaccine, inactivated, neutralizing antibody, T cell response, B cell response

## Abstract

In the past few years, the continuous pandemic of COVID-19 caused by SARS-CoV-2 has placed a huge burden on public health. In order to effectively deal with the emergence of new SARS-CoV-2 variants, it becomes meaningful to further enhance the immune responses of individuals who have completed the first-generation vaccination. To understand whether sequential administration using different variant sequence-based inactivated vaccines could induce better immunity against the forthcoming variants, we tried five inactivated vaccine combinations in a mouse model and compared their immune responses. Our results showed that the sequential strategies have a significant advantage over homologous immunization by inducing robust antigen-specific T cell immune responses in the early stages of immunization. Furthermore, the three-dose vaccination strategies in our research elicited better neutralizing antibody responses against the BA.2 Omicron strain. These data provide scientific clues for finding the optimal strategy within the existing vaccine platform in generating cross-immunity against multiple variants including previously unexposed strains.

## 1. Introduction

Coronavirus disease-19 (COVID-19) caused by SARS-CoV-2 has overwhelmed healthcare systems globally [1]. As the virus evolves, vaccines need to evolve unceasingly. As of 30 March 2023, 183 COVID-19 vaccines were in the clinical phase, while 199 were in pre-clinical development [2]. These vaccines are divided into DNA-based, RNA-based, protein subunit, virus-like particles (VLP), non-replicational viral vector, replicating viral vector, live-attenuated and inactivated vaccines [3,4] etc. Although these SARS-CoV-2 vaccines have played a huge role in efforts to bring the COVID-19 pandemic under control, it is still a great challenge for us to build a safe, future-proofed, durable immune repertoire to protect against the rapidly forthcoming viral variants.

Thus far, two or three doses of a homologous vaccine immunization strategy have already been used worldwide [5,6]. China began work on a fourth-dose vaccination program in December 2022 [7]. Many countries and regions, including Israel, Italy, Singapore, Denmark, Chile and others, also have launched a fourth-dose strategy already [8,9]. In addition, Japan, Cambodia and others have conducted fifth-dose vaccination strategies. Furthermore, to better cope with the variable SARS-CoV-2 variants, “heterologous prime-boost” strategies have been introduced in COVID-19 vaccination [10,11]. Heterologous booster vaccine strategy refers to the use of vaccine boosters from different technology platforms in order to improve the immune responses and enhance protection against variants [12]. Some studies have already shown that the heterologous prime-boost strategies can indeed improve the immunogenicity and efficacy of the vaccine [13,14] and elicit elevated virus-specific neutralizing antibody magnitude and breadth [15,16]. Most of the approved COVID-19 vaccine antigens are designed based on the spike protein of SARS-CoV-2 prototype strain revealed in Wuhan in 2020, there are still few studies using vaccines based on different variants for sequential immunization. Moreover, recent studies show that “original antigenic sin” may exist in COVID-19 infection, as the infection history of the original virus strain seems to “interfere” with the normal immune response to Omicron infection [17,18,19,20,21]. It is critical to understand whether the sequential booster derived from seasonal VOC sequences can break the “original antigenic sin” and induce better immunity against forthcoming variants.

CoronaVac (inactivated SARS-CoV-2 vaccine) (Sinovac Biotech Co., Beijing, China) is one of inactivated vaccines against COVID-19, approved for emergency use by the World Health Organization (WHO) on 1 June 2021 [22,23]. By the end of 2022, more than 60 countries, regions and international organizations were using CoronaVac for large-scale vaccination; the global cumulative supply exceeded 2.9 billion doses [24]. Some studies have shown that, the seropositive rates were above 90.0% on day 28 after both the second and third doses, and the third-dose regimen of CoronaVac can significantly improve the level of neutralizing antibodies, with no serious adverse reactions related to the vaccine [25,26,27]. Furthermore, compared with CoronaVac-only regimens, when CoronaVac vaccines are administered before or after vaccines from different platforms, the heterologous schedule shows enhanced immunogenicity [28,29]. But we do not know much about the immune parameters of the immunization with different virus sequences on the same inactivated vaccine platform. Some noteworthy studies confirm that the exposure to different viral strains may induce a more diverse B cell repertoire in human influenza vaccination [30], and the inactivated Omicron vaccine could recall immune responses to the HB02, Omicron, and Delta viruses after one or two doses of BBIBP-CorV [31], suggesting that the sequential immunization of variant vaccine might have protective efficacy against SARS-CoV-2 VOCs.

As inactivated vaccines have a high vaccination rate and is relatively safe and well tolerated in a wider range of populations, we explored the utility of several homologous and sequential regimens with CoronaVac and Gamma strain-based inactivated vaccine in a mouse model. Our study aimed to provide a scientific basis and novel evidence for future, optimized vaccine approaches that generate potent cross-immunity against variant.

## 2. Materials and Methods

### 2.1. Cells, Viruses, Vaccines and Animals

Vero cells were applied for virus amplification and titer assessment. SARS-CoV-2 Prototype/Gamma (P1)/Omicron(BA.2) strains used in this study were provided by Sinovac Biotech Co., Beijing, China. SARS-CoV-2 inactivated vaccines employed in this study included Prototype strain and Gamma strain (Sinovac Biotech Co., Beijing, China) (Table 1). Female BALB/c mice aged 42–48 days (Sipeifu Biotechnology Co., Ltd., Beijing, China) were group-housed and fed with a general diet. The study was reviewed and approved by the experimental animal ethics review committee of Sinovac, and performed in accordance with relevant guidelines and regulations (YF-SOP-RD-Z01, 1 June 2021).

### 2.2. Immunizations

BALB/c mice (n = 10 per group) in two- or three-dose vaccine groups were immunized with 0.5 mL COVID-19 vaccines each time by intraperitoneal (I.P.) injection every 2 weeks. We designed 5 groups: 2 × Prototype strain vaccine, 2 × Gamma strain vaccine, 3 × Prototype strain vaccine, 3 × Gamma strain vaccine, 2 × Prototype strain vaccine + 1 × Gamma strain vaccine. PBS was utilized as negative control (Table 2).

### 2.3. Enzyme-Linked Immunosorbent Assay (ELISA)

Serum samples from mice were collected every 4 weeks (week 8, 12, 16) after the last immunization, and SARS-CoV-2 receptor-binding domain (RBD) and nucleocapsid (N) binding antibody titers were evaluated by ELISA. In brief, 96-well ELISA plates were coated with 2 μg/mL of recombinant SARS-CoV-2-Prototype/BA.2/BF.7 RBD protein and SARS-CoV-2-Prototype N protein at 4 °C overnight. Plates were washed with PBS containing 0.05% Tween-20 (PBS-T) and blocked with 5% non-fat milk in PBS (5% milk/PBS) for 1 h at RT. After the plates were washed, mice serum samples were serially diluted from 1/800 in two-fold dilutions in 5% milk/PBS and were added to each well before incubation at RT for 2 h. After washing, anti-mouse IgG-HRP (1:1000, Southernbiotech) was added to each well and incubated for 1 h. The TMB substrate was then added, and the reaction was stopped by adding 1 M H_2_SO_4_. Absorbance was read at 450nm and 630nm. ELISA endpoint titers were defined as the highest reciprocal serum dilution that yielded an absorbance 2-fold greater than background values.

### 2.4. IFN-γ Enzyme-Linked Immunospot Assay (IFN-γ ELISPOT)

The T cell responses of vaccinated mice were analyzed using a mouse IFN-γ ELISPOT set (BD Bioscience, San Jose, USA) following the manufacture’s protocol. Briefly, 100 µL of diluted Anti-IFN-γ solution was added to each well of an ELISPOT plate and stored at 4 °C overnight. Plates were washed with blocking solution (RPMI 1640 with 10% Fetal Bovine Serum, 1% Penicillin-Streptomycin-L-Glutamine) and blocked for 2 h at RT. After incubation, blocking solution was discarded, then 5 × 10^5^ splenocytes and SARS-CoV-2 S1/N peptide pools (1 µg/mL) were added per well. We incubated the plates for 18 h at 37 °C in a 5% CO_2_ and humidified incubator. The cell suspension was aspirated and washed extensively, then diluted biotinylated IFN-γ antibody was added per well and incubated for 2 h at RT. After the wells were washed, the diluted enzyme conjugate (Streptavidin-HRP) was added to each well and incubated for 1 h at RT. After incubation, wells were washed extensively, and 100 µL of Final Substrate Solution was added to each well. After monitoring the spot development for about 15 min, we washed the wells with deionized water to stop substrate reaction. We enumerated spots by ELISPOT plate reader (AID Gmbh, Strassberg, Germany).

### 2.5. Flow Cytometry

Antigen-specific T and B lymphocyte immune responses were characterized by flow cytometry. In brief, mouse spleens were removed under aseptic conditions after immunization, and splenocytes were added to the plate (1 × 10^6^ cells/well). Dead cells were excluded using the violet LIVE/DEAD stain kit (Invitrogen, Carlsbad, CA, USA). Then, cells were stained with antibodies for anti-CD3 BV711, anti-CD4 BV605, anti-CD19 BV510, anti-CD45 AF700, anti-PD-L2 PE/Dazzle 594, anti-CD185 PE, anti-PD-1 APC/Cyanine7, anti-CD44 FITC, anti-CD38 APC, anti-IgM BV421, anti-IgD BV650, anti-B200 PECy7, anti-I-A/I-E PECy5, and anti-CD138 PercpCy5.5 surface markers (Biolegend, San Diego, CA, USA) in dark for 20 min at RT. After washing, 200 µL of 2% polyformaldehyde (Sigma, St. Louis, MO, USA) was added to each well to fix the cells. Flow cytometric analyses were performed on a BD FACSLSR Fortessa instrument, and data were analyzed with FlowJo (TreeStar, Ashland, OR, USA).

### 2.6. Authentic SARS-CoV-2 Neutralization Assay

The neutralizing activity of mouse serum was assessed by SARS-CoV-2 micro-neutralization assay. The heat-inactivated serum was two-fold serially diluted in 96-well plate and added to the same volume of Prototype/Gamma/Omicron BA.2 (100 CCID50) virus strain solution, then incubated for 2 h at 37 °C in a 5% CO_2_ incubator. Vero cells were added to the mixture and cultured at 37 °C for 4 days. After incubation, the cells were inspected under inverted light microscopy for cytopathic effect (CPE) to assess the inhibitory activity of each dilution. Data were reported as 50% inhibitory doses (ID50), the highest dilutions of serum capable of completely preventing virus-induced CPE in at least 50% of the wells.

### 2.7. Statistical Analysis

Statistical analyses were performed with GraphPad Prism software (Prism V8.0.2). Comparisons between multiple groups were analyzed by one-way analysis of variance (ANOVA). A *p* value < 0.05 was considered statistically significant.

## 3. Results

### 3.1. The Third Vaccination Elicits Higher Levels of Binding Antibodies

**Figure 1:**
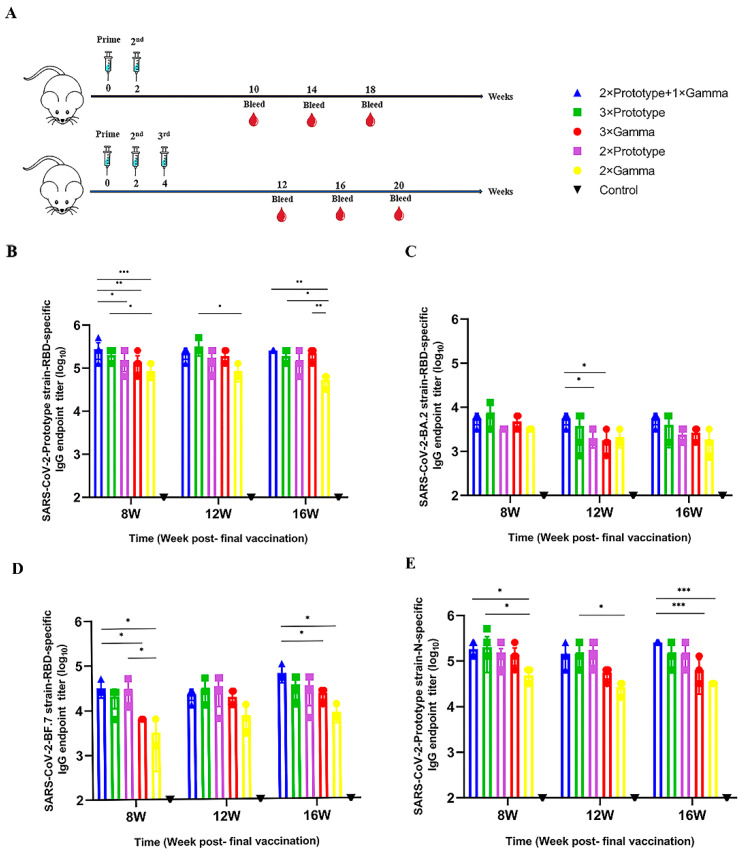
Comparison of SARS-CoV-2-RBD/N specific binding IgG titers of different COVID-19 vaccine regimens. (**A**) Schematic representation of experimental protocols and immunization groups. SARS-CoV-2 (**B**) Prototype, (**C**) BA.2, (**D**) BF.7 RBD-specific binding IgG titers and (**E**) Prototype N-specific binding IgG titers were measured by ELISA. Bars represent means ± SD, * *p* < 0.05, ** *p* < 0.01, *** *p* < 0.001.

To determine whether third vaccination-induced antibody responses can be enhanced, we tested five different vaccination strategies, and mice serum samples were collected at weeks 8, 12 and 16 after the final dose for SARS-CoV-2 RBD/N-based ELISA (Figure 1A). We evaluated anti-SARS-CoV-2-Prototype/BA.2/BF.7 RBD IgG antibody titers and anti-SARS-CoV-2-Prototype N IgG antibody titers by ELISA. The results showed that in contrast to the control group, all vaccinated groups could induce significantly higher levels of binding antibody. Meanwhile, mice inoculated with the same kind of vaccine showed a trend toward higher levels of SARS-CoV-2-Prototype RBD-specific IgG antibody in the three-dose group compared to the two-dose group. At 8, 12 and 16 weeks post immunization, the IgG titers of the 3 × Prototype group were higher than those of the 2 × Prototype group by 1.3, 1.8 and 1.3 fold, respectively, while the IgG titers of the 3 × Gamma group were higher than those of the 2 × Gamma group by 1.6, 2.3 and 4.2 fold, respectively (*p* < 0.01). The IgG antibodies of the 2 × Prototype group + 1 × Gamma group were 1.8, 1.3, and 1.8 times as high as those of the 2 × Prototype group, 3.2, 2.6, and 4.8 times as high as those of the 2 × Gamma group, 1.4, 0.7, 1.3 times as high as those of the 3 × Prototype group, and 2, 1.2, and 1.1 times as high as those of the 3 × Gamma group, respectively. Moreover, the SARS-CoV-2-Prototype RBD-specific IgG titer of the sequential-group mice also had some advantages compared to the other experimental groups. Additionally, the IgG levels of mice in each immune group did not change much over time, but remained relatively stable until the 16th week (Figure 1B). BA.2, BF.7 RBD-specific binding IgG titers and Prototype N-specific binding IgG titers also exhibited a similar trend, as described above (Figure 1C–E).

### 3.2. Durable and Cross-Reactive Neutralizing Antibody Responses Induced by the Third Vaccination

**Figure 2:**
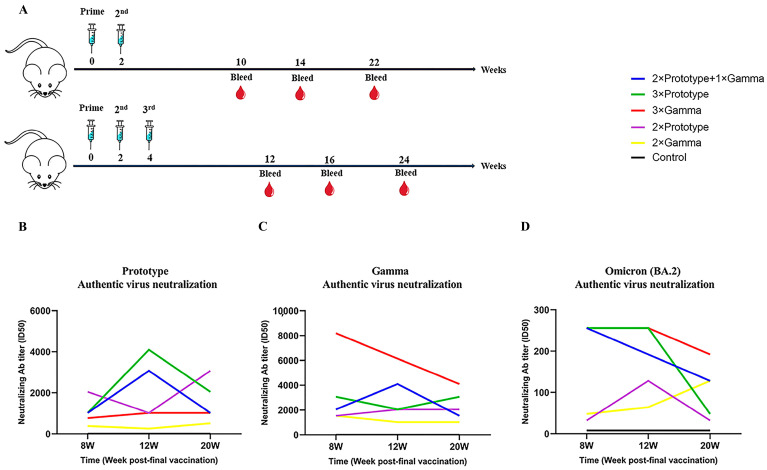
Comparison of humoral immune responses induced by COVID-19 vaccines of different regimens. (**A**) Schematic representation of experimental protocols and immunization groups. Mice serum samples at 8, 12 and 20 weeks post immunization were collected. Neutralizing antibody titers in serum against (**B**) Prototype (**C**) Gamma (**D**) Omicron BA.2 authentic strains.

The titers of anti-SARS-CoV-2-neutralizing antibodies against Prototype, Gamma and Omicron BA.2 strains in sera of vaccinated mice were measured using authentic SARS-CoV-2-micro-neutralization assays. Mice serum samples were collected at weeks 8, 12 and 20 after the final dose (Figure 2A). The results showed that the ID50 (50% inhibitory dose) against Prototype strain in mice receiving 3 × Prototype and 2 × Prototype + 1 × Gamma was higher than that of the other groups (Figure 2B). Likewise, the serum ID50 against Gamma strain in mice receiving 3 × Gamma and 2 × Prototype + 1 × Gamma was higher than that mice in the other groups at 8 to 20 weeks post last vaccination (Figure 2C). It is worth mentioning that the serum samples of mice in these five vaccination groups could also neutralize the COVID-19 variant Omicron (BA.2). Although the neutralization antibody titer to Omicron (BA.2) did drop substantially, the serum ID50 of mice receiving three doses of vaccine administration was still higher than that of mice receiving two doses (Figure 2D).

### 3.3. Activation of Memory B Cells by the Third Vaccination

**Figure 3:**
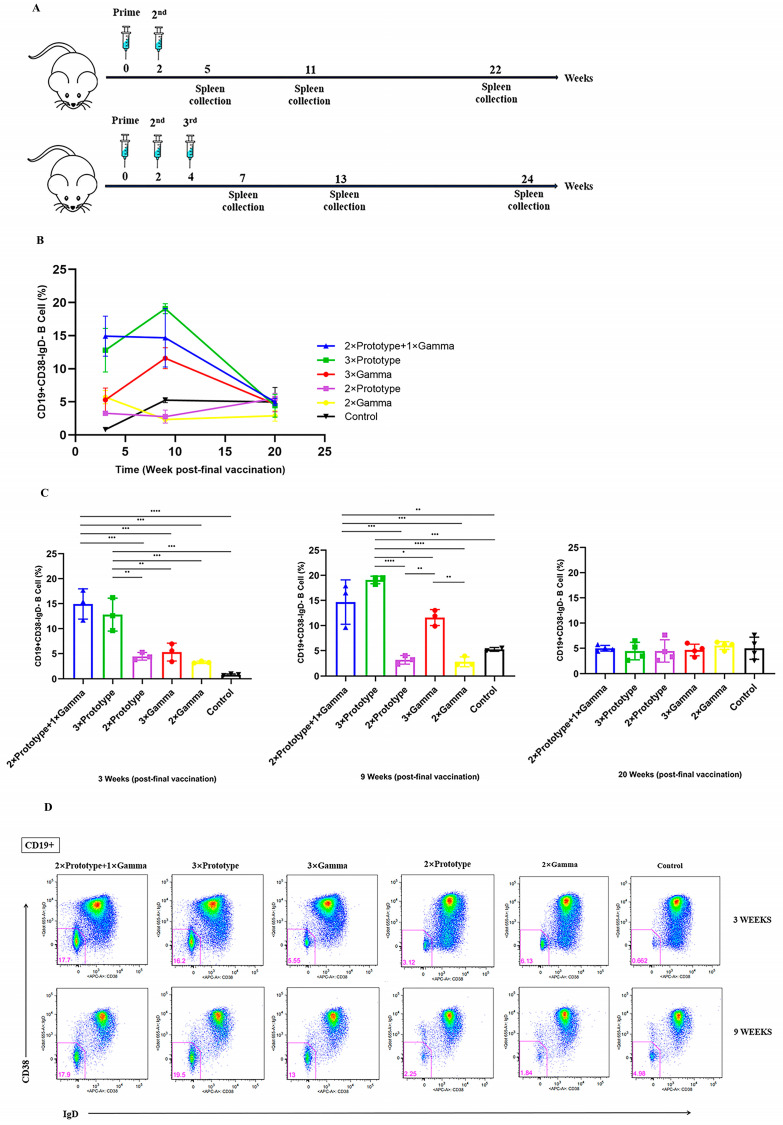
Comparison of memory B cells induced by COVID-19 vaccines of different regimens. (**A**) Schematic representation of experimental protocols and immunization groups. (**B**,**C**) Frequency of CD19^+^CD38^−^IgD^−^ memory B cells measured by flow cytometry. (**D**) The CD19^+^CD38^−^IgD^−^ memory B cells in the 3rd and 9th week after immunization, respectively. * *p* < 0.05,** *p* < 0.01, *** *p* < 0.001, **** *p* < 0.0001.

To further explore the humoral response induced by the vaccination, the frequencies of different B cell subsets were evaluated by flow cytometry. Mouse spleens were acquired at 3, 9 and 20 weeks after final immunization (Figure 3A). As shown in Figure 3B, the frequency of CD19^+^CD38^−^IgD^−^ memory B cells in the three-dose groups (2 × Prototype + 1 × Gamma, 3 × Prototype, 3 × Gamma) was higher than in the two-dose vaccine groups (2 × Prototype, 2 × Gamma) at the 3rd and 9th week after the last vaccination. At the 3rd and 9th week, the proportion of CD19^+^CD38^−^IgD^−^ memory B cells in the 3 × Prototype group was higher than that in the 2 × Prototype group (*p* < 0.01 and *p* < 0.0001 respectively), and the 3 × Gamma group had a greater proportion of memory B cells than the 2 × Gamma group (*p* < 0.01). The proportion of CD19^+^CD38^−^IgD^−^ memory B cells in the 2 × Prototype + 1 × Gamma group was higher than that in the 2 × Prototype (*p* < 0.001), 2 × Gamma (*p* < 0.001), 3 × Gamma (*p* < 0.001) and 3 × Prototype groups at the 3rd week, and higher than that of the 2 × Prototype (*p* < 0.001), 2 × Gamma (*p* < 0.001) and 3 × Gamma groups at the 9th week. In the 20th week, almost no difference were found among the groups (Figure 3C,D).

### 3.4. Higher SARS-CoV-2-Specific T Cell Responses Elicited by Sequential Immunization

**Figure 4:**
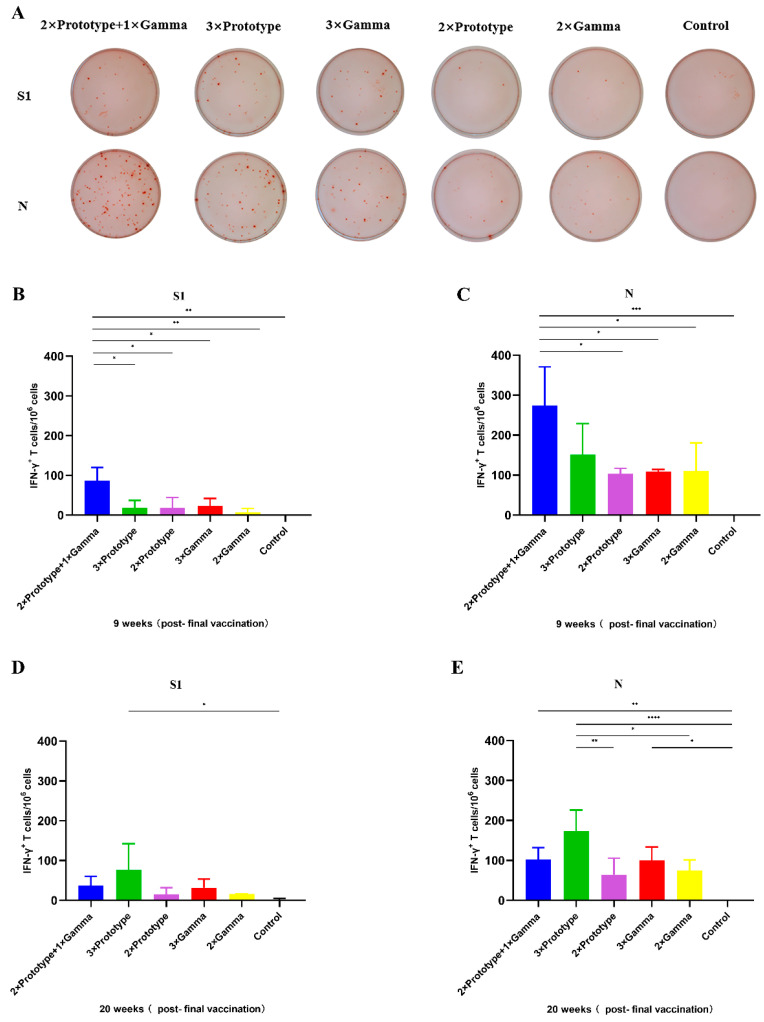
SARS-CoV-2 specific T-cell immune responses in immunized mice. (**A**) Qualitative representation of ELISpot results. (**B**,**C**) Magnitude of SARS-CoV-2-specific cell responses directed against S1 and N regions. IFN-γ was evaluated by an ELISpot assay from a pool of splenocytes derived from immunized mouse groups at 9 weeks post vaccination. (**D**,**E**) Magnitude of SARS-CoV-2-specific cell responses directed against S1 and N regions. The splenocytes were derived from immunized mouse groups at 20 weeks post vaccination. Bars show triplicate mean values and the standard deviation. * *p* < 0.05, ** *p* < 0.01, *** *p* < 0.001, **** *p* < 0.0001.

To evaluate the adaptive SARS-CoV-2-specific T-cell immune responses, splenocytes were stimulated by spike protein S1 subunit (S1) and N peptide pools and were measured by ELISpot. The results demonstrated that, compared to the control group, both the three-dose and two-dose groups could elicit significantly higher levels of IFN-γ^+^ T cells against SARS-CoV-2 S1 and N peptide pools. More importantly, sequential immune groups could stimulate the strongest S1 and N protein immune responses at 9 weeks post vaccination (Figure 4A). T cells secreting IFN-γ from 2 × Prototype + 1 × Gamma group mice were more numerous than those from other groups (Figure 4B,C). At 20 weeks post vaccination, 3 × Prototype immune groups could stimulate the strongest S1 and N protein immune responses; next was the 2 × Prototype + 1 × Gamma group (Figure 4D,E). Moreover, the level of IFN-γ^+^ T cells against SARS-CoV-2 S1 and N peptide in the 3 × Prototype group was higher than that in the 2 × Prototype group, and the level in the 3 × Gamma group was also higher than that in the 2 × Gamma group.

## 4. Discussion

By the end of January 2023, China had approved the use of 13 COVID-19 vaccines, five of which were inactivated vaccines. Among the inactivated vaccines, CoronaVac and Covilo have been widely used in China and several other countries [33]. By the end of January 2023, China had administered 3.49 billion doses of COVID-19 vaccines, with inactivated vaccines accounting for the majority. Although many clinical trials have confirmed that they have good safety and can prevent COVID-19 [11,34,35], it is still a huge challenge to face the new, emerging SARS CoV-2 variants in the future. The emergence of Omicron was first identified on 24 November 2021 in South Africa and quickly overtook the last VOC (Delta variant) as the predominant lineage [36]. Optimizing the strategy based on vaccination with the prototype antigen to obtain immunity against the variants was not only required to deal with the Omicron strains at that time, but is also needed in order to deal with new variants in the future.

CoronaVac is a chemically inactivated whole virus SARS-CoV-2 vaccine that has been proved to be well tolerated and safe in individuals and provides a good humoral response to SARS-CoV-2. The plasma neutralizing antibodies elicited by CoronaVac suggest that the third-dose vaccination may be beneficial for combating SARS-CoV-2 variants, such as 501Y.V2 (B.1.351) and Omicron (B.1.1.529) [23,37]. To overcome the influence of pre-existing antibodies on the effect of subsequent vaccination, WHO, with support from the Strategic Advisory Group of Experts (SAGE) and its COVID-19 vaccines work group, has been evaluating evidence on the use of heterologous boosting schedules [13]. In addition, different vaccines can complement each other. Enhancing vaccine effectiveness, increasing immunogenicity and reducing reactogenicity are the advantages of heterologous vaccination [12]. At present, heterologous product combinations are diverse, but there are few reports about sequential immunization using different SARS-CoV-2 variants-based inactivated vaccines. It is necessary to characterize both the humoral and cellular response as well as neutralization capacity of sequential regimens with inactivated vaccines.

Antibodies against the S protein, especially the receptor-binding domain (RBD) epitope, are essential for preventing the virus from entering target cells. Accumulating evidence suggests that binding antibody (IgG) response plays an important role in protection against SARS-CoV-2 infection [38]. Our results showed that the three-dose immune regimens improved the SARS-CoV-2-prototype-RBD IgG level slightly compared to the two-dose regimens. Besides that, the SARS-CoV-2-prototype-RBD IgG binding antibody titer of the three-dose sequential immunization program was the highest at 8 and 16 weeks post immunization. The results were consistent with those of previous studies [39,40,41] and also suggested that the binding antibody responses of each immune group were very stable and not much different from the 8th to 16th week. It is noteworthy that all vaccine groups can produce SARS-CoV-2 variant (BA.2/BF.7)-RBD specific IgG binding antibodies, and the antibody levels can be maintained for a period of time. Nucleocapsid is an indispensable part of viral transcription, replication, and participation in cellular regulation [42]. Compared to RBD, there are fewer mutations in the N protein. So, in this study, we only tested the SARS-CoV-2-Prototype-N specific IgG, and the results showed that it has trend characteristics similar to those of RBD specific IgG.

To further explore the neutralizing-antibody response induced by these immune strategies of inactivated SARS-CoV-2 vaccines and to understand whether these strategies could provide cross-neutralization to VOC, we assessed cross-neutralizing activity against Prototype, Gamma and Omicron (BA.2) variants in mice serum after the final dose. Our results showed that the neutralizing ability of three-dose immunized mice was higher than that of two-dose immunized mice, consistent with existing research results [43]. The neutralizing antibody response elicited by all of these immune regimens in mice can neutralize the SARS-CoV-2 Prototype, Gamma and Omicron (BA.2) variants. What is more noteworthy is that after three doses, both sequential immunization and homologous immunization can better induce mice to produce neutralizing antibodies against the Omicron BA.2 strain. Although the level of neutralizing antibody decreased, it could also provide a certain degree of cross-neutralizing activity. In addition, the 3 × Gamma group had the strongest ability to neutralize the Gamma strain, and the 2 × Prototype + 1 × Gamma group also had a good neutralizing effect on the Gamma strain. Most of the neutralization antibodies declined after 8 weeks, which is consistent with previous studies [44,45]. Several studies have shown that the neutralizing antibody increases rapidly from the 14th to 28th day after immunization, and then gradually decreases. On this basis, we speculate that in the future, sequential immunization by current VOC- or dominant variant-based vaccines may be able to produce better humoral immunity against variants.

SARS-CoV-2 spike (S) protein specific memory B cells detected in recovered individuals are considered to be related to virus neutralization and have protective value [46,47,48]. For this reason, memory B cells are considered as a key component of long-lived immunity against SARS-CoV-2 infection [49,50]. Here, we analyzed the memory B cell phenotypes in mice with different immunization programs. Results showed that the proportion of CD19^+^CD38^−^IgD^−^ memory B cells in mice inoculated with three doses of inactivated vaccine was higher. Some previous studies also have shown that the third dose can elicit increased numbers of memory B cells, and these memory B cells can express more potent and broader antibodies [51,52,53]. It is worth mentioning that the proportion of memory B cells induced by sequential immunization is significantly higher than that of other vaccine groups in terms of promoting humoral immunity especially at the early stage after last vaccination. Besides that, there were almost no differences in the frequency of CD19^+^CD38^−^IgD^−^ memory B cells among groups in the 20th week after immunization, which means that the immune effect produced by the vaccine was already weak. This phenomenon is consistent with the study results of Chen et al. [54]. This conclusion supports the hypothesis that the humoral responses are long-lasting and contribute to future recall responses.

Understanding the role of T cell immunity in vaccine-mediated SARS-CoV-2 protection is another important mechanism for future vaccine design and understanding the susceptibility of COVID-19. In previous studies, T cell responses have been suggested to play an important role in protecting against SARS-CoV-2 [55,56,57]. SARS-CoV-2 has four key structural proteins: the nucleocapsid (N) protein, spike (S) protein, matrix (M) protein, and envelope (E) protein [58]; not only S protein, but also N protein has high immunogenicity and can induce a strong cellular immune response [59,60]. Among the SARS-CoV-2 vaccines, the mRNA vaccines and other technical route vaccines only use S protein as the target, however, inactivated vaccines contain the whole virus particle. In addition to S protein, inactivated vaccines also contain N protein, M protein, etc., which makes them more likely to break the “original antigenic sin”. The experiment results published by the National Institutes of Health (NIH) and other research groups show that mRNA-1273 or mRNA-Omicron boost in vaccinated macaques elicits similar B cell expansion, neutralizing responses, and protection from Omicron [61], the effect of the new mRNA-Omicron is limited. In our study, the results of SARS-CoV-2-specific T cell immune responses showed that mouse from 2 × Prototype + 1 × Gamma group in the 9th weeks elicited higher levels of IFN-γ secreting T cells against both SARS-CoV-2 S1 and N peptide pools. This finding informed us that sequential regimens with inactivated vaccines of different virus sequences have advantages in enhancing cellular immune response, which may due to the conserved N proteins. The limitation of this study is that only the Gamma strain inactivated vaccine was used for sequential vaccination. In the future, the latest Omicron strains or VOCs should be considered for research on immunization strategies.

In summary, our study characterized the humoral and cellular responses evoked by five COVID-19 inactivated vaccination strategies in a mouse model. The three-dose inactivated vaccine regimens induced higher immune responses compared to the two-dose counterparts in mice. Importantly, our results provide scientific data in support of the three-dose COVID-19 inactivated vaccine and confirmed that the sequential regimens with inactivated vaccines based on different variant sequences can achieve stronger cellular immune response. Our study also further confirmed that, in concert with robust memory humoral immunity, the preferential induction of N-specific T cell response may indicate a key hallmark of antiviral immunity that is likely to confer cross-protection against emerging SARS-CoV-2 variants.

## Figures and Tables

**Table 1:** Information concerning vaccines used in this study.

Abbreviation	Type of Vaccine	Vaccine Platform Description	Dose (SU/mL)
Prototype strain vaccine	COVID-19 Vaccine (Vero cell), Inactivated CoronaVac [32]	Inactivated virus	1200
Gamma strain vaccine	COVID-19 Vaccine (Vero cell), Inactivated, Gamma strain	Inactivated virus	1200

SU: virus antigen contents.

**Table 2:** Schematic representation of immunization groups.

Group	1st	2nd	3rd
Negative control	PBS	PBS	PBS
2 × Prototype + 1 × Gamma	Prototype strain vaccine	Prototype strain vaccine	Gamma strain vaccine
3 × Prototype	Prototype strain vaccine	Prototype strain vaccine	Prototype strain vaccine
3 × Gamma	Gamma strain vaccine	Gamma strain vaccine	Gamma strain vaccine
2 × Prototype	Prototype strain vaccine	Prototype strain vaccine	
2 × Gamma	Gamma strain vaccine	Gamma strain vaccine	

## Data Availability

Not applicable.
